# How Common Is Femoroacetabular Impingement Morphology in Asymptomatic Adults? A 3D CT-Based Insight into Hidden Risk

**DOI:** 10.3390/jcm14207220

**Published:** 2025-10-13

**Authors:** Pelin İsmailoğlu, Cengiz Kazdal, Emrehan Uysal, Alp Bayramoğlu

**Affiliations:** 1 Department of Anatomy, Faculty of Medicine, Recep Tayyip Erdogan University, Rize 53020, Turkey; 2 Department of Orthopaedics and Traumatology, Faculty of Medicine, Recep Tayyip Erdogan University, Rize 53020, Turkey; cengiz.kazdal@erdogan.edu.tr; 3 Independent Researcher, Faculty of Medicine, Recep Tayyip Erdogan University, Rize 53020, Turkey; emrehan_uysal24@erdogan.edu.tr; 4 Department of Anatomy, Faculty of Medicine, Acibadem Mehmet Ali Aydinlar University, Istanbul 34752, Turkey; alp.bayramoglu@acibadem.edu.tr

**Keywords:** femoroacetabular impingement, alpha angle, lateral center-edge angle, three-dimensional CT, asymptomatic hip morphology

## Abstract

**Background and Objectives**: Femoroacetabular impingement (FAI) morphology refers to structural abnormalities that can alter normal joint mechanics and potentially lead to early onset osteoarthritis. Although commonly diagnosed in symptomatic individuals, such morphological features are also found in asymptomatic adults, underlining their relevance for early detection and preventive management. This study aimed to evaluate the three-dimensional congruence of hip joint surfaces in relation to FAI and the morphology of asymptomatic hips with potential FAI features. **Materials and Methods**: Retrospective three-dimensional reconstructions of 86 hip joints were created using Mimics software from computed tomography (CT) scans of the lower abdomen and pelvis retrieved from the radiology archive. CT scans belonged to individuals with preserved anatomical integrity (20 females, 23 males, bilateral hips), aged 24–76 years. Lateral center-edge angle (LCEA) and alpha angle measurements were obtained from reconstructions to assess the risk of asymptomatic FAI. **Results**: Significant gender differences were found in alpha angles. The mean right alpha angle was 46.57 ± 3.12° in females and 49.28 ± 6.66° in males *p* = 0.046, while the mean left alpha angle was 43.75 ± 5.53° in females and 47.37 ± 5.77° in males *p* = 0.021. An alpha angle >50°, suggestive of cam type FAI, was present in 25.6% of right hips and 13.9% of left hips. LCEA values showed no significant gender or side differences, with a mean of 30.21 ± 8.96° across the cohort. **Conclusions**: Three-dimensional evaluation of asymptomatic hips revealed FAI-consistent morphology in a notable proportion of individuals, particularly males. Cam-type deformities tended to occur bilaterally, whereas pincer-type morphologies were more sporadic and often unilateral. Increased alpha and LCEA measurements in asymptomatic individuals suggest that FAI morphology may exist subclinically without always indicating disease. Future studies incorporating longitudinal imaging and clinical follow-up are needed to clarify the prognostic significance of these findings.

## 1. Introduction

Femoroacetabular impingement (FAI) is a mechanical hip disorder that arises from abnormal contact between the femoral head–neck junction and the acetabular rim, often leading to cartilage and labral damage and eventually early-onset osteoarthritis (OA) in young adults [1,2]. FAI is commonly classified into three morphological types: cam, pincer, and mixed, each characterized by distinct structural deformities [2,3]. Cam-type lesions typically result from an aspherical femoral head that increases the alpha angle (the radiological measure of femoral head sphericity), while pincer-type lesions involve excessive acetabular coverage, evaluated using the lateral center-edge angle (LCEA), which reflects the degree of acetabular overcoverage [3,4].

Although FAI has traditionally been investigated in symptomatic populations, numerous studies have shown that characteristic morphological findings may also be present in asymptomatic individuals [1,5]. In these cases, standard imaging parameters—particularly alpha and LCEA measurements—play a critical role in identifying subclinical deformities that may progress to symptomatic disease [3]. The presence of FAI morphology in asymptomatic individuals poses a significant clinical challenge, as early recognition of these anatomical variations could enable timely interventions and possibly reduce the burden of late-stage degenerative hip disorders [4].

In recent years, three-dimensional computed tomography (3D-CT) has emerged as a valuable modality for evaluating FAI-related bony parameters with high anatomical precision [2,6]. It allows for comprehensive morphometric analysis of the hip joint, contributing to a better understanding of the prevalence and characteristics of FAI morphology even in individuals without symptoms. Despite its diagnostic advantages, studies investigating FAI morphology in asymptomatic adults using 3D-CT in the Turkish population remain limited [1,3,7].

Several clinically relevant questions remain unresolved. Although FAI morphology can exist without symptoms, it is unclear why some individuals develop pain and functional limitation while others with similar anatomical features do not [6]. Can asymptomatic individuals with morphologic findings be considered at true risk, or are these variations simply benign? The literature lacks clarity on whether alpha and LCEA measurements can predict symptom development and whether asymptomatic cases with such findings should be managed proactively. To address these uncertainties and better understand the clinical significance of FAI-related morphologies in asymptomatic populations, this study focuses on characterizing the distribution of FAI morphology in asymptomatic adults.

Specifically, we evaluated alpha and lateral center-edge angles using retrospective 3D-CT data from asymptomatic individuals to assess the prevalence of FAI morphology. By focusing on this population, our findings offer insight into its distribution and potential implications for early detection and preventive care strategies.

## 2. Materials and Methods

This retrospective observational cross-sectional study was conducted using lower abdominal and pelvic computed tomography (CT) scans obtained from the radiology archive during the study period between 2017 and 2019. These scans were originally acquired for clinical indications unrelated to hip pathology. A total of 150 lower abdominal and pelvic CT scans were screened. To include anatomically intact hips with preserved bony anatomy, patients with pelvic trauma, orthopedic conditions, or pelvic malignancy were excluded. All participants had no documented history of hip pain, trauma, or surgery. CT scans had been acquired for non-orthopedic clinical indications such as gastrointestinal assessments. As a result, 43 asymptomatic adults (23 males, 20 females; aged 24–76 years) were included if images demonstrated adequate quality for 3D reconstruction. Eligible cases were selected consecutively from the archive to minimize selection bias. Ethical approval for the study was granted by the Clinical Research Ethics Committee of Acıbadem University and Acıbadem Healthcare Institutions Medical Research Ethics Committee (ATADEK) (approval number: 2020-01/43), and all data were anonymized prior to analysis.

All CT scans were acquired in the supine position using a Somatom Force scanner (Siemens Healthineers, Erlangen, Germany) with standard protocols (100 kVp, 256 mAs), a slice thickness of 0.5–1.0 mm, and an image matrix of 512 × 512 pixels (voxel size ≈ 0.7 × 0.7 × 0.5 mm^3^). The scans were reconstructed with a standard bone algorithm and converted into three-dimensional (3D) models using Mimics^®^ software (Materialise, Leuven, Belgium). These 3D reconstructions were used for detailed morphological evaluations to assess the presence or potential risk of femoroacetabular impingement (FAI) in an asymptomatic population. In each case, the two key morphometric parameters—lateral center-edge angle (LCEA) and alpha angle—were measured bilaterally to evaluate anatomical predisposition.

Quality control procedures were performed at two stages: (1) image selection, where only high-quality scans with preserved anatomical detail were accepted, and (2) measurement, where images with reconstruction or angle measurement difficulties were excluded prior to final analysis. As a result, no measurement failures occurred in the final dataset. All measurements were performed by two independent blinded observers. Observer 1 (7 years of clinical experience in hip pathology) completed the initial set of measurements and repeated them approximately six months later to assess intraobserver reliability. Observer 2 (9 years of expertise in musculoskeletal morphology) independently repeated all measurements in a blinded manner to evaluate interobserver reliability.

### 2.1. Morphometric Analysis

#### 2.1.1. Lateral Center-Edge Angle (LCEA)

**Figure 1:**
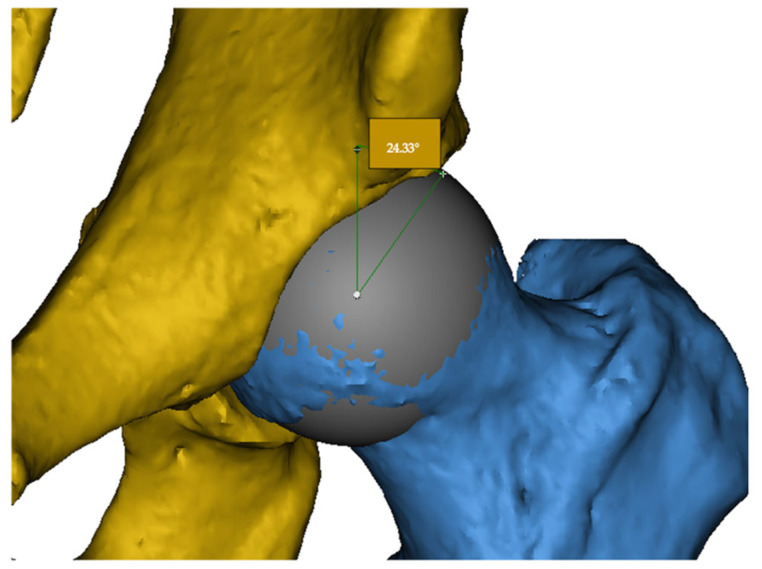
Measurement of the lateral center-edge angle (LCEA) on a 3D CT reconstruction. The acetabulum is shown in yellow and the femoral head in blue. The example demonstrates an LCEA of 24.33°.

The LCEA is defined as the angle between a vertical line passing through the center of the femoral head and a line drawn from the center to the lateral edge of the acetabular roof. This angle quantifies the degree of acetabular coverage over the femoral head. Excessive LCEA values suggest pincer-type impingement, whereas low values may indicate acetabular dysplasia (Figure 1).

#### 2.1.2. Alpha Angle

**Figure 2:**
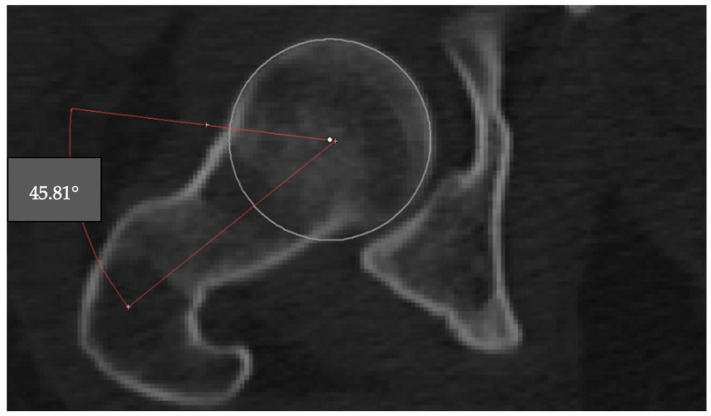
Measurement of the alpha angle on an axial CT slice. The angle is formed between the femoral neck axis and a line drawn from the femoral head center to the point where the head contour deviates from the best-fit circle (45.81°).

The alpha angle is used to assess the sphericity of the femoral head and detect cam-type deformities. The angle was measured on the axial CT slice where the femoral head appeared largest. A best-fit circle was drawn around the subchondral bone of the femoral head, and two lines were drawn: one from the center of the femoral head through the neck axis, and another from the center to the point where the femoral head extended beyond the circular contour. The angle between these two lines was recorded as the alpha angle (Figure 2).

For the interpretation of the measurements, specific threshold values were utilized. A lateral center-edge angle (LCEA) below 20 degrees was considered indicative of acetabular undercoverage, whereas values between 20 and 40 degrees were accepted as representing a normal hip joint [8,9]. An LCEA greater than 40 degrees was interpreted as suggestive of pincer-type femoroacetabular impingement [8,9]. In terms of the alpha angle, values up to 42 degrees were considered within normal limits, with no expected impingement [9]. An alpha angle exceeding 50 degrees was regarded as pathological in Western populations, and values over 60 degrees were indicative of cam-type femoroacetabular impingement (Table 1) [3].

### 2.2. Statistical Analysis

Mean values for LCEA and alpha angles were calculated. Measurements were compared between sexes and sides (right vs. left) using appropriate statistical tests, including the independent samples *t*-test for normally distributed variables and the Mann–Whitney U test for non-normally distributed data to assess for any significant anatomical differences. To ensure adequate statistical power, an a priori power analysis was conducted at the study’s planning stage using G*Power 3.1. Based on a paired *t*-test, a medium effect size (Cohen’s d = 0.50), α = 0.05, and a desired power of 0.95, the analysis indicated that a sample size of *n* = 45 would be sufficient. Post hoc power analysis, conducted after data collection using observed effect sizes for the right (d = 0.509, power = 94.9%) and left (d = 0.639, power = 99.3%) alpha angles, confirmed that the study was adequately powered [10].

To assess measurement reliability, inter- observer and intra-observer analyses were performed. Previously obtained measurements were re-evaluated for this purpose. Measurements were repeated six months later for intra-observer assessment, while an independent evaluation of the alpha angle and LCEA was conducted for inter-observer assessment. Both inter- and intra-observer reliability were quantified using the Intraclass Correlation Coefficient (ICC). All analyses were conducted in SPSS Statistics Version 26.0 (IBM Corp., Armonk, NY, USA). A *p*-value < 0.05 was considered statistically significant. In addition to conventional statistical tests, effect size calculations (Cohen’s d) were performed to assess the magnitude and clinical relevance of the observed differences.

Data Availability Statement: The CT scan data analyzed in this study were obtained from the hospital’s medical imaging archive and are not publicly available due to patient confidentiality restrictions. Anonymized data may be available from the corresponding author upon reasonable request and with permission from the institutional review board.

Generative AI Statement: No generative artificial intelligence (GenAI) tools were used in the preparation of this manuscript beyond superficial language editing (grammar, spelling, punctuation, and formatting).

## 3. Results

### 3.1. Participant Characteristics

A total of 43 asymptomatic individuals (20 females [46.5%], 23 males [53.5%]) with a total of 86 hips (bilateral) were included in this study. The age of participants ranged from 24 to 76 years, with a mean of 49.44 ± 16.58 years.

### 3.2. Measurement Reliability

To evaluate measurement reliability, intra- and inter-observer analyses were performed separately, and agreement was assessed using intraclass correlation coefficients (ICC). Intra-observer reliability analysis demonstrated high agreement for all angular measurements, with ICC values of 0.976 (95% confidence interval (CI): 0.951–0.988) for right LCEA, 0.951 (95% CI: 0.911–0.973) for left LCEA, 0.984 (95% CI: 0.971–0.992) for right alpha angle, and 0.975 (95% CI: 0.955–0.987) for left alpha angle (all *p* < 0.001). Inter-observer reliability analysis also showed high levels of agreement, with ICC values of 0.947 (95% CI: 0.674–0.982) for right LCEA, 0.989 (95% CI: 0.979–0.994) for left LCEA, 0.941 (95% CI: 0.893–0.968) for right alpha angle, and 0.995 (95% CI: 0.987–0.998) for left alpha angle (all *p* < 0.001). The ICC analysis confirmed that the angular measurements were reliable and reproducible, indicating that the findings are consistent and not substantially influenced by measurement error.

### 3.3. Alpha Angle Measurements

The alpha angle values demonstrated a significant difference between genders. The mean right alpha angle was higher in males (49.28° ± 6.66) compared to females (46.57° ± 3.12), with a *p*-value of 0.046, indicating statistical significance. Similarly, the left alpha angle was significantly greater in males (47.37° ± 5.77) than in females (43.75° ± 5.53), with a *p*-value of 0.021 (Table 2).

When alpha angle values were categorized, 23.3% of the right hips and 11.6% of the left hips had values in the 50–60° range, while 2.3% of hips on both sides exceeded 60°. These thresholds are typically associated with pathological cam-type morphology. Despite the absence of symptoms, these morphological changes were notably present, especially in males.

### 3.4. Lateral Center-Edge Angle (LCEA) Measurements

No statistically significant differences were observed in LCEA values between males and females. However, males had consistently higher LCEA values than females on both sides. The mean right LCEA was 33.22° ± 5.28 in males and 30.21° ± 8.96 in females, while the left LCEA was 32.20° ± 6.35 in males and 29.74° ± 6.52 in females (Table 2).

In terms of distribution, 14% of right hips and 4.7% of left hips demonstrated an LCEA greater than 40°, which is typically associated with pincer-type femoroacetabular impingement. Conversely, LCEA values below 20° were observed in 7% of right hips and 2.3% of left hips, suggestive of undercoverage or acetabular dysplasia.

Effect size analyses (Cohen’s d) demonstrated small differences for the lateral center-edge angle (LCEA) (right: −0.416, 95% CI −1.019 to 0.193; left: −0.383, 95% CI −0.985 to 0.225). For the alpha angle, the effect sizes were slightly larger, indicating small-to-moderate differences (right: −0.509, 95% CI −1.115 to 0.104; left: −0.639, 95% CI −1.250 to −0.021). Overall, these results suggest that the observed differences reflect modest clinical effects.

### 3.5. Correlation Analysis

To address the potential interdependencies between variables, we conducted correlation analyses for LCEA, alpha angle, and age for both hips. The results indicated no significant correlations between these parameters in our sample. Specifically, for the right hip, the correlation between LCEA and alpha angle (Spearman’s rho) was not significant (r = −0.039, *p* = 0.804), and for the left hip the correlation between LCEA and alpha angle (Pearson) was not significant (r = −0.133, *p* = 0.395). There was no correlation between age and the angular measurements (LCEA and alpha angle) in our sample.

There was no significant correlation between alpha angle and LCEA measurements. Pearson’s correlation coefficient between right alpha angle and right LCEA was r = −0.022 (*p* = 0.888), while between left alpha angle and left LCEA, r = −0.133 (*p* = 0.395), suggesting an independent variation in these morphometric parameters.

### 3.6. Summary of Findings

As shown in Table 2, alpha angle values demonstrated gender-specific differences, with males exhibiting higher values bilaterally. Although LCEA values did not differ significantly between genders, a proportion of asymptomatic individuals displayed values above or below clinical thresholds. These findings highlight the presence of femoroacetabular impingement (FAI) morphology in individuals without clinical symptoms.

As presented in Table 3, the prevalence and type distribution of asymptomatic FAI were determined based on angular thresholds defined in the current literature [3,8,9]. A total of 8 hips (8.5%) demonstrated a pincer-type FAI pattern (LCEA > 40°), while 2 hips (2.1%) showed alpha angles greater than 60°, indicative of cam-type FAI. Additionally, 15 hips (15.9%) had alpha angles exceeding 50°, which are considered pathological in Western populations, suggesting potential anatomical predisposition to cam-type FAI [3,8,9].

None of the cases in this study demonstrated combined cam- and pincer-type morphology; each finding was observed in isolation without overlap.

## 4. Discussion

Femoroacetabular impingement (FAI) is increasingly recognized as a morphological abnormality that, if left undetected, can contribute to the development of early-onset osteoarthritis despite the absence of symptoms [5]. The identification of FAI-related bony features such as an increased alpha angle and altered acetabular coverage has gained particular importance in asymptomatic individuals [5,11]. In a CT-based study by Koirala et al., alpha angle and acetabular version were evaluated in young asymptomatic individuals, emphasizing the potential risk these morphologies pose even in the absence of symptoms [11]. Similarly, Cengiz et al. reported that a substantial portion of asymptomatic adults exhibited alpha and center-edge angle values above pathological thresholds, suggesting latent joint incongruity with possible long-term consequences [1]. However, the scarcity of 3D CT-based analyses in Turkish cohorts leaves important questions unanswered regarding the true distribution and characteristics of FAI morphology in this population. Furthermore, Mascarenhas et al. demonstrated in a 3D CT-based analysis that hip morphology is gender-specific yet bilaterally symmetric, reinforcing the need for individualized anatomical evaluation in early FAI detection [12]. These findings collectively highlight the importance of early radiological screening to support preventive strategies that may delay or avert degenerative hip disorders [5,11,12].

This study evaluated the three-dimensional morphological characteristics of asymptomatic hips using CT-based alpha and lateral center-edge angles (LCEA) to estimate the possible occurrence—rather than the exact prevalence—of FAI morphology. A significant proportion of asymptomatic individuals, particularly males, demonstrated alpha angle values suggestive of cam-type deformities. The presence of cam-type morphology (alpha angle > 50°) in 27.9% of hips, pincer-type (LCEA > 40°) in 9.3%, and mixed-type in 2.3% aligns with findings in previous reports. The cutoff values applied were consistent with both international standards and recent Turkish studies [3,8,9], supporting their appropriateness for interpreting FAI morphology in this population. Mascarenhas et al. similarly reported that cam morphology occurs in a notable proportion of asymptomatic cases, reinforcing our observation that structural changes can exist without symptoms [9,13]. Additionally, Koirala et al. emphasized the importance of detecting subclinical morphologies using CT for early diagnosis [11]. Our findings also reinforce the hypothesis proposed by Zadpoor, where repetitive biomechanical loading during adolescence may lead to morphologic changes in the femoral head–neck junction, particularly in active individuals [14]. This may partly explain the higher cam prevalence in males, possibly due to lifestyle or anatomical predispositions [15]. In line with this, the recent sports medicine literature has also emphasized how repetitive mechanical loading and mobility demands influence lower extremity biomechanics in active populations. For instance, a randomized controlled trial demonstrated that integrative balance and plyometric training improved balance, ankle mobility, and jump performance in youth football players, highlighting the link between repetitive loading, mobility, and potential risk factors for joint pathology [16]. Such findings support our interpretation that morphological variations observed in asymptomatic hips may, in part, reflect adaptive responses to cumulative biomechanical stress, particularly in younger or athletic individuals.

Although radiographic evidence of FAI morphology is common, many individuals remain asymptomatic [11]. This raises questions about the necessity and timing of surgical intervention [6]. The current study’s results suggest that the mere presence of FAI morphology in asymptomatic individuals should be interpreted as a potential risk factor rather than a definitive disease state. Furthermore, surgical correction is not invariably required; conservative approaches such as physiotherapy and activity modification may be sufficient in many cases, particularly when symptoms are absent or mild. Recent evidence from a multilevel meta-analysis by Ramadanov et al. further supports this perspective, demonstrating that conservative treatment can provide comparable outcomes to hip arthroscopy in selected patients with femoroacetabular impingement [17]. Our results reinforce that morphological risk does not always equate to clinical pathology, echoing Neumann et al.’s observation that alpha angle correction can restore impingement-free motion without guaranteeing symptom improvement [9,18]. Clinically, it is essential to distinguish between morphological risk and symptomatic disease [5,19]. It has also been noted that timely treatment of FAI not only addresses ongoing symptoms but may also mitigate progressive cartilage and labral damage, thereby reducing long-term risk of osteoarthritis [18]. As noted by Cengiz et al. and Tosun et al., conservative options such as physiotherapy can be effective, especially for mild or asymptomatic findings [1,20]. The high rate of asymptomatic morphology supports further research into the predictive value of imaging for early osteoarthritis prevention [3,6]. Our use of 3D CT allowed for more precise evaluation of alpha and LCEA measurements, confirming that males tend to have higher alpha angles, consistent with both Turkish and international studies [1,3,7,8]. Although the absolute differences in alpha angle and LCEA between genders were modest (2–4°), these differences consistently reached statistical significance and are in line with previous reports describing sex-related variation in hip morphology [13]. Despite the small-to-moderate effect sizes, even subtle angular variations may influence hip biomechanics and contribute to long-term FAI risk, underscoring their potential clinical relevance [21,22].

From a biomechanical perspective, asymptomatic morphology on one side may affect contralateral hip mechanics due to the bilateral hip function [21,23]. Thus, unilateral intervention should be considered cautiously, as untreated contralateral morphology may contribute to later dysfunction [23]. In our study, no subject had isolated unilateral cam-type morphology with alpha > 60°, suggesting that when cam-type morphology are present, they are typically bilateral or absent [15,21]. In contrast, unilateral pincer-type morphology (LCEA > 40°) was observed in six cases—five right-sided and one left-sided—indicating that pincer-type variations may be more sporadic [12,13,22,23]. This finding underscores the importance of assessing both hips independently, as compensatory loading on the “normal” side may predispose it to future degeneration [23,24]. Even in unilateral symptomatic patients, latent risk factors on the contralateral side should be considered [5,9,12,23,25]. Longitudinal studies may be helpful to explore potential biomechanical, genetic, or environmental factors that could contribute to progression from asymptomatic morphology to clinical disease [26,27]. One notable finding of our study was the lack of a significant correlation between age and LCEA or alpha angle, despite the wide age range of our asymptomatic participants. This may reflect that morphological changes associated with FAI develop early in life and remain relatively stable over time or that compensatory mechanisms enable asymptomatic individuals to maintain hip biomechanics despite age-related changes. Further research is needed to explore the relationship between age and hip morphology in both asymptomatic and symptomatic populations.

A major challenge in the literature is the lack of standardization in pathological thresholds [3,8,11,27]. The alpha angle cut-off for cam-type FAI varies between >50° and >60°, depending on imaging method and population, complicating comparisons [3,8]. Similarly, LCEA interpretation differs between CT and plain radiography [10,24]. Variations in imaging modality (2D vs. 3D) and measurement techniques further limit inter-study consistency [2,3,11,13,15,23,27]. The 3D CT approach in our study offers a methodological advantage by providing detailed anatomical visualization and precise morphometric data. The alignment of our results with other Turkish cohorts and CT-based literature indicates that our findings are consistent with previous reports and may reflect general trends in this population.

By presenting new 3D morphometric data from an asymptomatic Turkish population, this study contributes to the limited evidence describing the distribution of FAI-related morphologies in this group. The observed frequency of subclinical FAI morphology provides descriptive information that may be relevant for understanding potential structural variations in the hip. These findings may offer preliminary insight into the possible role of subclinical hip morphology in early diagnostic and preventive research, while underscoring the need for further longitudinal studies to clarify its clinical significance.

### Limitations

This study has several limitations. Only two morphological parameters—alpha angle and LCEA—were evaluated. While widely used in FAI diagnosis, other measures such as femoral head–neck offset, acetabular version, and femoral version were not assessed. The focus on alpha angle and LCEA was intentional, as these parameters are among the most clinically validated and widely used in both national and international literature, allowing direct comparability with previous studies and ensuring methodological consistency. In addition, potential variability related to imaging protocols, software use, and observer expertise may have influenced the measurements. However, our inter- and intra-observer reliability analyses demonstrated high agreement, suggesting that the impact of such variability on the findings was minimal. Moreover, as this was an observational study without information on participants’ physical activity, causal inferences cannot be made, and the findings should be interpreted with caution. A further limitation is that labral integrity was not evaluated, although it may play an important role in explaining why some individuals with FAI-consistent morphology remain asymptomatic and could also be relevant to long-term osteoarthritis risk. Finally, the lack of long-term follow-up limits conclusions regarding progression; future prospective studies combining morphologic, functional, and clinical outcomes would be valuable.

## 5. Conclusions

This study highlights that a considerable proportion of asymptomatic adults present with alpha angle and lateral center-edge angle measurements suggestive of cam- or pincer-type morphologies when assessed using high-resolution three-dimensional computed tomography. Cam-type deformities were more prevalent in males and tended to occur bilaterally, whereas pincer-type morphologies were sporadic and often unilateral.

## Figures and Tables

**Table 1:** Threshold values for LCEA and alpha angle in femoroacetabular impingement (FAI) [3,8,9].

Parameter	Threshold Value	Interpretation
LCEA (Lateral Center-Edge Angle)	<20°	Acetabular Undercoverage [3,8,9]
LCEA (Lateral Center-Edge Angle)	20–40°	Normal Hip Joint [3,8,9]
LCEA (Lateral Center-Edge Angle)	>40°	Suggestive Of Pincer-Type FAI [3,8,9]
Alpha Angle	≤42°	Within Normal Limits [3,8,9]
Alpha Angle	>50°	Pathological İn Western Populations [3,8,9]
Alpha Angle	>60°	Indicative Of Cam-Type FAI [3,8,9]

**Table 2:** Mean Values of Alpha Angle and LCEA in Males and Females With Statistical Comparison thresholds in the present study.

Measurement	Male Mean ± Sd	Female Mean ± Sd	*p*-Value
Right Alpha Angle	49.28 ± 6.66	46.57 ± 3.12	0.046
Left Alpha Angle	47.37 ± 5.77	43.75 ± 5.53	0.021
Right LCEA	33.22 ± 5.28	30.21 ± 8.96	0.091
Left LCEA	32.20 ± 6.35	29.74 ± 6.52	0.109

**Table 3:** Classification of FAI based on LCEA and alpha angle thresholds in the present study.

Classification [3,8,9]	Number of Hips
LCEA < 20° (Acetabular Undercoverage)	4
20° ≤ LCEA ≤ 40° (Normal Hip Joint)	74
LCEA > 40° (Pincer-Type FAI)	8
Alpha ≤ 42° (Normal)	13
42° < Alpha ≤ 50° (Borderline)	56
Alpha > 50° (Pathological)	15
Alpha > 60° (Cam-Type FAI)	2

## Data Availability

We confirm that the main data supporting this study’s findings are available within the article, and any additional data are available upon request from the corresponding author.

## Abbreviations

The following abbreviations are used in this manuscript:

FAI	Femoroacetabular Impingement
3D	Three-dimensional
CT	Computed Tomography
LCEA	Lateral Center-Edge Angle
